# Early Experience of Trans-arterial Chemo-Embolisation for Hepatocellular Carcinoma with a Novel Radiopaque Bead

**DOI:** 10.1007/s00270-019-02317-3

**Published:** 2019-08-27

**Authors:** John Reicher, Sebastian Mafeld, Georgia Priona, Helen L. Reeves, Derek M. Manas, Ralph Jackson, Peter Littler

**Affiliations:** Newcastle Upon Tyne Hospitals NHS Foundation Trust, Newcastle Upon Tyne, UK

**Keywords:** TACE, Trans-arterial chemoembolisation, HCC, Hepatocellular carcinoma, LUMI

## Abstract

**Aims:**

To evaluate early outcomes of patients with hepatocellular carcinoma (HCC) treated with a novel radiopaque bead, the 75–150 μm DC Bead LUMI™ (Biocompatibles UK Ltd).

**Materials and Methods:**

This was a retrospective review of the first 40 consecutive patients at a UK tertiary hepato-biliary centre, treated for HCC with TACE using radiopaque beads, between May 2017 and March 2019. Information regarding complications, mortality, lesion response and subsequent ablation procedures was collected from electronic records and case notes. Intra- and post-operative imaging was reviewed for visibility of the embolised territory.

**Results:**

Fifty-five TACE procedures were performed in 40 patients, with a median age of 70 years (range 28–88) and median lesion size of 3.8 cm (range 1.5–7.8). The median follow-up period was 30 weeks (range 6–101). Mean post-procedure hospital stay was 1.2 days. Complications of CIRSE Grade II or above occurred after 4/55 procedures (7.3%). Mortality at 30 days was zero. Objective response rates (mRECIST) at 1, 3 and 6 months were 32/35 (91.4%), 21/24 (87.5%) and 12/15 (80%), respectively. Complete response rates at 1, 3 and 6 months were 16/35 (45.7%), 12/24 (50%) and 9/15 (60%). The embolised territory was visible on intra-operative and follow-up CT imaging in all patients. The radiopaque beads were used as a fiducial marker to guide ablation in 5/40 patients (12.5%).

**Conclusion:**

TACE with radiopaque beads shows promising tolerability and efficacy. The radiopaque beads ensure visualisation of the embolised lesion on intra- and post-operative imaging and, in selected cases, can act as a marker for CT-guided ablation.

## Introduction

Trans-arterial chemo-embolisation (TACE) is an established treatment option for intermediate-stage hepatocellular carcinoma (HCC) [1–3]. Two dominant approaches have been proposed, conventional TACE (cTACE) and drug-eluting bead TACE (DEB-TACE).

While neither has been shown to confer an overall survival advantage, DEB-TACE has the advantage of lower concentrations of chemotherapeutic agent reaching the systemic circulation, with fewer attendant side effects; however, some operators prefer cTACE due to the superior visibility on intra-operative imaging of ethiodised oil compared to the mixture of beads and contrast medium used in DEB-TACE [4–6]. Following cTACE, lipiodol may be retained by tumour cells for several months, and therefore the embolised lesion may be visualised on post-operative CT [7]; however, following DEB-TACE the contrast agent dissipates, meaning the embolised territory cannot be directly visualised.

DC Bead LUMI™ (Biocompatibles UK Ltd, UK) is a bead with iodine incorporated into its chemical structure, ensuring that it is permanently radiopaque [8]. This potentially combines the advantages of DEB-TACE, with low systemic drug absorption, and cTACE, with good visualisation on post-operative imaging. Despite this theoretically promising concept, little is known about the bead’s safety profile and efficacy [9–11]. This study evaluates early outcomes in the first cohort of patients treated with radiopaque beads at our institution.

## Materials and Methods

### Patient Cohort

We conducted a retrospective review of the first 40 consecutive patients who underwent TACE for HCC with DC Bead LUMI™ at our institution, a tertiary hepato-biliary referral centre in the UK. The study period was May 2017–March 2019. Patient demographics, underlying aetiology, BCLC tumour stage and liver function, were obtained from the clinical notes, electronic patient record and picture archiving and communication system (PACS). In all cases, HCC was diagnosed according to current European Association for Study of Liver guidelines [3] and all patients were deemed suitable for TACE by a multi-disciplinary tumour board. All procedures were performed in accordance with the ethical standards of the institutional research committee and with the 1964 Helsinki Declaration and its later amendments [12].

Complications were recorded and classified according to the CIRSE system [13]. Follow-up imaging was reviewed for evidence of response in the treated lesions, according to mRECIST [14]. For patients who underwent more than one TACE, the follow-up period was deemed to start on the date of the first treatment. Patient survival was documented until April 30, 2019.

### Follow-Up Imaging Protocol

Standard imaging follow-up was quadruple-phase CT (pre-contrast, arterial, portal venous and delayed venous phases) at 1- and 3-month post-TACE, and then at 3-month intervals thereafter. MRI was reserved mainly for lesions with equivocal response on CT, given relatively limited local availability of MRI.

### Outcome Measures

Primary outcome measures were TACE-related complications, lesion response at 1, 3 and 6 months after the initial procedure and 30-day mortality.

Two radiologists reviewed imaging for all patients, including intra-operative fluoroscopy and (if available) cone-beam CT (CBCT) from each patient’s first TACE procedure with radiopaque beads, and follow-up pre-contrast CT imaging at 1 and 12 months, to determine whether the embolised territory (the HCC itself and/or the peri-tumoral vessels) could be clearly identified.

We recorded all patients who subsequently underwent ablation procedures, noting whether the radiopaque beads were used as a fiducial marker to aid placement of the ablation needle under CT guidance.

### Exclusion Criteria

Four patients who underwent additional treatments for HCC in the first month following TACE (3 underwent thermal ablation and 1 liver transplantation) were excluded from analysis of treatment response.

### DC Bead LUMI™ Technical Considerations

The DC Bead LUMI™ is available in two sizes—70–150 μm and 100–300 μm. The core chemical structure is the same as the DC Bead™, a polyvinyl alcohol-2-acrylamido-2-methylpropane sulphonic acid hydrogel bead. To create the DC Bead LUMI™, an iodine-containing moiety—2,3,5-triiodobenzaldehyde—is added to the PVA backbone.

As well as rendering it radiopaque, incorporation of this moiety affects the bead’s physical properties in two additional ways: its density (1.3 g/cm^3^) is 30% greater and its compressibility is two orders of magnitude lower compared to the DC Bead™. The increased density results in more rapid sedimentation; therefore, it is recommended that DC Bead LUMI™ is mixed with a high-viscosity contrast agent to maintain a workable suspension and microcatheters flushed with contrast agent rather than saline to ensure bead clearance [8].

Unlike the DC Bead™, which shrinks between 20 and 30% when loaded with doxorubicin, the DC Bead LUMI™ remains the same size following drug loading. Its drug loading and elution kinetics are similar to the DC Bead™ [8].

### Doxorubicin-Loading and Dosage Protocol

Beads were loaded with doxorubicin by in-house pharmacy staff according to protocols developed in conjunction with the manufacturers. Dosage was in line with published recommendations, i.e. planned doses of up to 75 mg doxorubicin loaded onto one 2-ml vial of beads if the disease was within the Milan criteria for liver transplantation, or up to 150 mg doxorubicin loaded onto two vials of beads for more advanced disease [15].

### Procedure Details

All patients received broad-spectrum antibiotic prophylaxis for 3 days, starting on the day of the procedure.

All procedures were carried out using common femoral artery access. In all cases, digital subtraction angiography and CBCT were used to map the tumour vessels and allow selective or super-selective embolisation, aiming for an endpoint of pre-stasis. In 25/55 procedures, post-embolisation CBCT was performed to evaluate completeness of tumour coverage; this was dependent on factors including radiation dose, lesion characteristics and operator preference.

In 53/55 cases, a 2.7Fr progreat microcatheter (Terumo Corporation, Japan) was used; a steerable SwiftNINJA™ microcatheter (Merit Medical, USA) with a 2.4Fr tip was used in one patient with tortuous anatomy, and a 2.0Fr progreat microcatheter was used in one case with small tumour vessels.

The smaller bead size (75–150 μm) was used in all cases. Beads were mixed with high osmolarity contrast agent, typically omnipaque 350 mg/ml (GE Healthcare, USA), at a ratio of 2-ml beads (i.e. one vial) to 18 ml contrast agent. Just prior to administration, the mixture was agitated between two syringes to maintain a uniform suspension and injected using controlled pulses from a 2-ml syringe at an approximate rate of 1 ml/min under direct fluoroscopic screening.

## Results

### Patient Cohort

Fifty-five TACE procedures with DC Bead LUMI™ were carried out in 40 patients (37 men and 3 women), a mean of 1.4 procedures per patient. The median age was 70 years (mean 68.6, range 28–88). The median lesion size was 3.8 cm (mean 4.0, range 1.5–7.8). Disease characteristics are summarised in Table 1.

**Table 1:** Basic disease characteristics

Aetiology of liver disease	
Non-alcoholic fatty liver (NAFLD)	14 (35%)
Alcoholic liver disease (ALD)	9 (22.5%)
Mixed	3 (7.5%)
Hepatitis C	4 (10%)
Other/unknown	8 (20%)
No background liver disease	2 (5%)
Cirrhosis	
Cirrhotic	28 (70%)
Non-cirrhotic	12 (30%)
Histological diagnosis	
Well differentiated HCC	10 (25%)
Moderately differentiated HCC	10 (25%)
Poorly differentiated HCC	3 (7.5%)
No histology (radiological diagnosis)	17 (42.5%)
Number of lesions	
1 lesion	31 (77.5%)
2 lesions	7 (17.5%)
3 lesions	2 (5%)
Size of target lesion (or largest lesion if multiple)	
≤ 2 cm	3 (7.5%)
2.1–3 cm	10 (25%)
3.1–4 cm	11 (27.5%)
4.1–5 cm	7 (17.5%)
> 5 cm	9 (22.5%)
BCLC stage	
Stage A	17 (42.5%)
Stage B	23 (57.5%)
Child–Pugh score	
A	39 (97.5%)
B	1 (2.5%)

### Adverse Events and Complications

All 55 procedures were completed as planned with no intra-operative complications. Thirty-day mortality was zero. Minor complications requiring no additional therapy or deviation from the standard post-operative course (i.e. CIRSE Classification Grade 1) [13] were recorded after eight procedures—six patients reported post-procedure discomfort and two reported minor groin bruising.

Post-operative complications which required additional treatment or further hospital stay (i.e. CIRSE Grade 2 or above) occurred after four procedures, as summarised in Table 2.

**Table 2:** Summary of post-procedure complications requiring additional treatment or hospital stay

	Complication	Treatment required	CIRSE classification [14]
1	Post-embolisation syndrome (fever)	Inpatient stay extended by 72 h for monitoring	Grade 3
2	Post-embolisation syndrome (malaise, fever)	Attended hospital for unplanned clinical review. Discharged with advice	Grade 2
3	Post-embolisation syndrome (pain, fever)	48-h admission for symptomatic treatment	Grade 3
4	Liver abscess	Re-admitted day 13 with newly diagnosed CBD stones, and liver abscess in the segment treated by TACE, requiring IV antibiotics and percutaneous drainage. No permanent sequelae	Grade 3

The first three documented complications were in patients who had relatively high-volume disease (lesions ≥ 4.5 cm). The fourth—a liver abscess in the embolised liver segment—occurred in a patient who also had obstructing common bile duct (CBD) calculi with intrahepatic duct dilatation. This was not detected on the pre-TACE imaging, blood tests or clinical examination.

### Lesion Response Rates

Lesion response rates are summarised in Table 3. After exclusion criteria were applied, 1-month follow-up was available for 35 patients, 3-month follow-up for 24 patients and 6-month follow-up for 15 patients. Objective response was calculated by adding complete response and partial response.

**Table 3:** Lesion response on early post-treatment imaging according to mRECIST

	1 month	3 months	6 months
Number of patients with data available	35	24	15
Complete response	16(45.7%)	12 (50%)	9 (60%)
Partial response	16 (45.7%)	9 (37.5%)	3 (20%)
Objective response	32(91.4%)	21 (87.5%)	12 (80%)
Stable disease	2 (5.7%)	1 (4.2%)	0
Progressive disease	1 (2.9%)	2 (8.3%)	3 (20%)

### Survival

38/40 patients (95%) remain alive, with a median follow-up period of 6 months (range 1–23). 2/40 patients (5%) have died during the follow-up period. The first (male, 71 years) had an initial TACE procedure with radiopaque beads for two right-lobe HCCs (3.8 cm and 4.0 cm), with complete response in both lesions on imaging up to 6 months. The CT at 9 months showed recurrence in one of the lesions, which was targeted with a second TACE procedure at 10 months, achieving a partial response at 11 months. Subsequent imaging showed progressive disease. He was deemed unsuitable for further TACE or medical therapy. He received supportive care and died 10 months later, 21 months after the first TACE.

The second death occurred in a 63-year-old man treated for a 5.0-cm moderately differentiated HCC, with a background of Child–Pugh A alcohol-induced cirrhosis. His 1-month follow-up scan post-TACE showed stable disease; at 3 months, there was recurrence in the treated lesion and new lesions elsewhere in the liver. He subsequently developed ascites and encephalopathy and died 5 months after the TACE procedure.

### Imaging

Both radiologists reviewing the imaging agreed that in all 40 patients, the embolised territory was clearly visible on intra-operative fluoroscopy. Post-embolisation CBCT was performed in 21/40 patients (52.5%) at their first TACE procedure, and in all cases the embolised territory was clearly visible. In 39/39 patients with 1-month follow-up CT imaging available, the embolised territory was clearly visible on the unenhanced scan, regardless of whether or not there had been an intervening ablation procedure. In 10/10 patients with 12-month follow-up CT imaging available, the embolised territory remained clearly visible on the unenhanced scan.

MRI was used in addition to CT for follow-up imaging in 10/40 patients (25%).

### Ablation

In the follow-up period, 6/40 patients (15%) subsequently underwent microwave ablation of lesions initially treated by TACE. This included four patients with HCC under 3 cm in diameter, who underwent ablation within 6 weeks of TACE as a pre-planned second stage of treatment. One patient had a 3-month scan showing partial response following TACE of a 3-cm lesion and had an ablation targeting the area of residual enhancement. One patient underwent CT-guided ablation of a small nodule of local recurrence 18 months after the initial TACE (discussed below).

In five of six patients who underwent ablation, tumour staining with radiopaque beads was used as a fiducial marker to assist needle placement under CT guidance. In the other patient, the lesion was targeted with ultrasound. All patients treated with ablation showed no evidence of local recurrence in the follow-up period.

## Discussion

### Safety

A previous study involving over 400 DEB-TACE treatments suggests that some degree of post-embolisation syndrome is a near-universal feature, with more severe post-embolisation symptoms reported in 27.1% of patients [16]. While comparison with our results is challenging given the differences in data collection, we observed post-embolisation syndrome requiring further hospital stay or medical assessment after only 3/55 procedures (5.5%).

Regarding the other significant complication we observed, the obstructing CBD calculi are thought to have played a major role in the development of the liver abscess. Had the biliary obstruction been appreciable on pre-operative evaluation, TACE would have been postponed until after duct clearance.

### Efficacy

Given this study’s retrospective design, absence of a control group and relatively short follow-up periods for many of the patients, analysis of tumour response is limited. Any comparison of outcomes with other studies should be approached with caution given potential differences in study design, patient selection and TACE technique. However, early response rates in our cohort fall within the expected range, given previously reported peak response rates following DEB-TACE of between 44 and 99.5% [5, 16, 17].

### Intra-operative Imaging

**Fig. 1:**
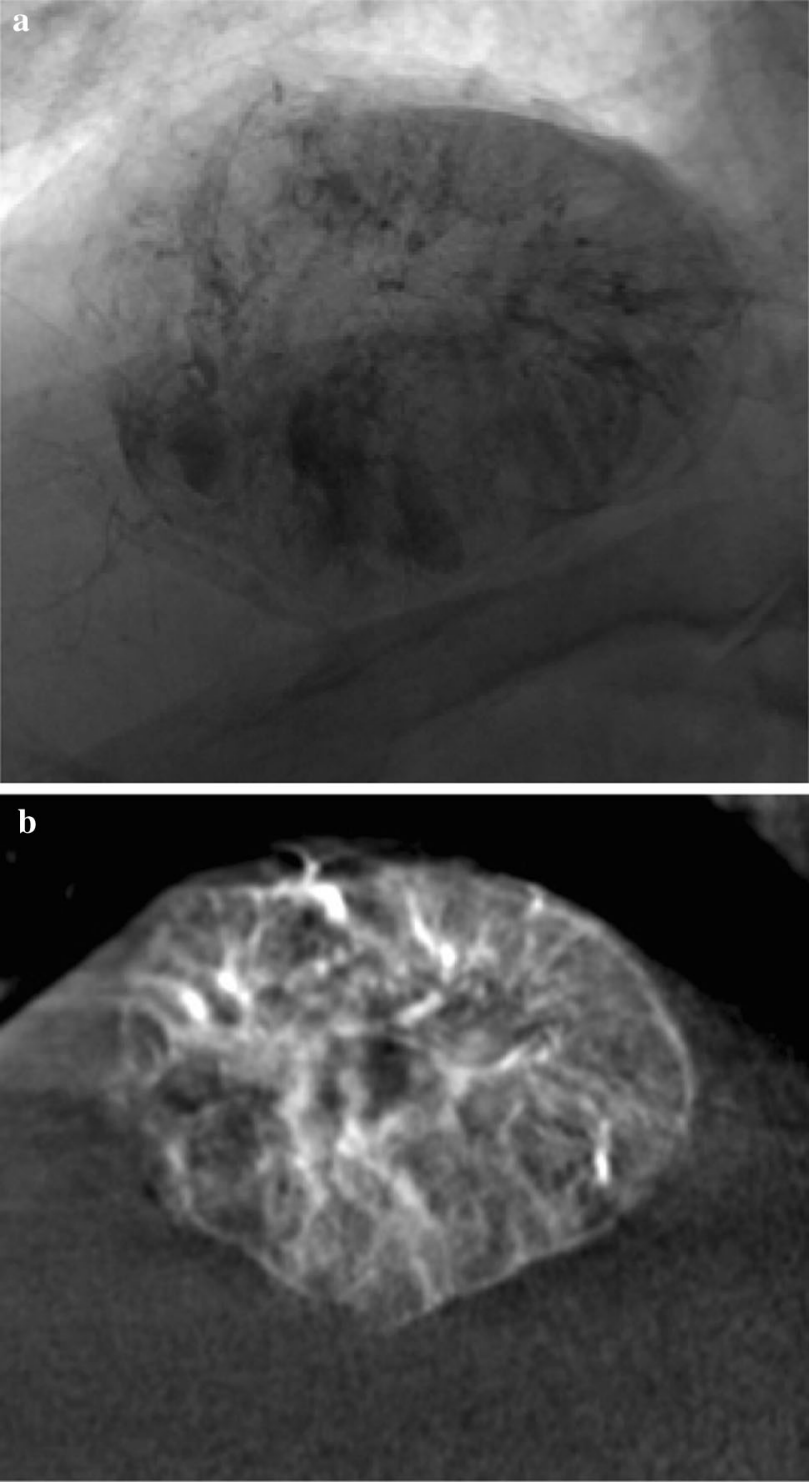
Intra-operative imaging. **a** a fluoroscopic image and **b** a coronal image from cone-beam CT, both taken at the end of the procedure, demonstrating tumour staining with a combination of radiopaque beads and trapped contrast medium

Our findings demonstrate that the radiopaque beads allow the embolised territory to be clearly seen intra-operatively, both on fluoroscopy and CBCT, an example is shown in Fig. 1. Good intra-operative visualisation means that non-target embolisation can be recognised, potentially allowing catheter repositioning to minimise the associated risks.

### Follow-Up Imaging

**Fig. 2:**
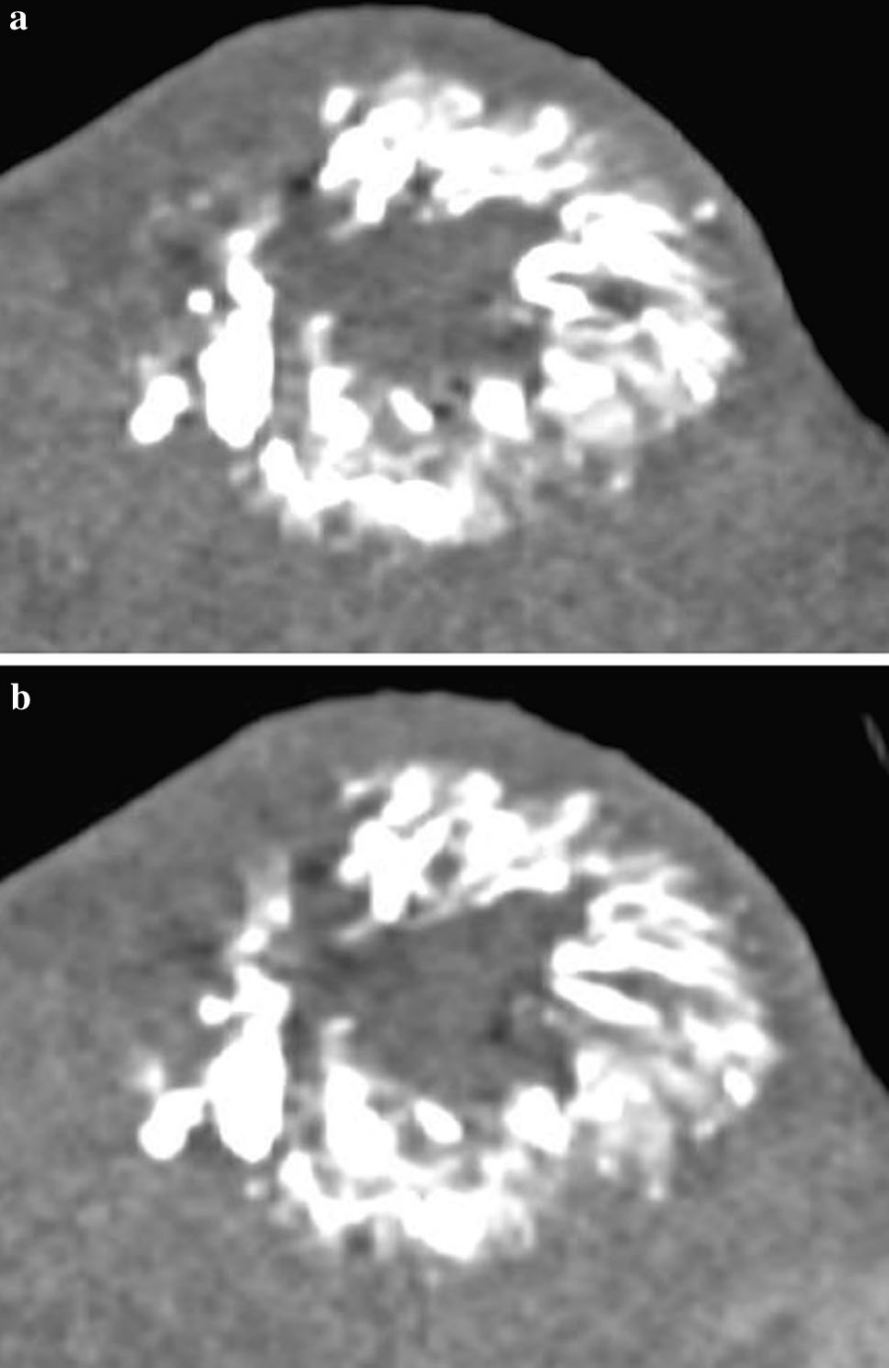
Post-operative imaging, noncontrast (Fig. 2a) and arterial phase (Fig. 2b) coronal images from the 1-month follow-up CT from the same patient as Fig. 1, demonstrating persistent tumour staining with radiopaque beads. There was no postcontrast enhancement, indicating a complete response according to mRECIST

We have demonstrated that the embolised territory following treatment with radiopaque beads remains visible on follow-up CT imaging (Fig. 2). Consequently, radiologists involved in reporting follow-up imaging must be made aware of the differences in expected appearances following TACE with radiopaque beads, in order to reduce the potential for misinterpretation. For example, hyper-density in the embolised territory on pre-contrast imaging should be recognised as staining by radiopaque beads and not mistaken for post-treatment calcification. Contrast-enhanced MRI, used in one quarter of our cohort, may be helpful if follow-up CT is equivocal.

### Tumour Marking for Ablation

We have demonstrated that the beads’ visibility on follow-up CT imaging can mark lesions for ablation under CT guidance. Given that the beads remain visible after ablation, post-treatment margins can also be assessed on subsequent CT imaging.

**Fig. 3:**
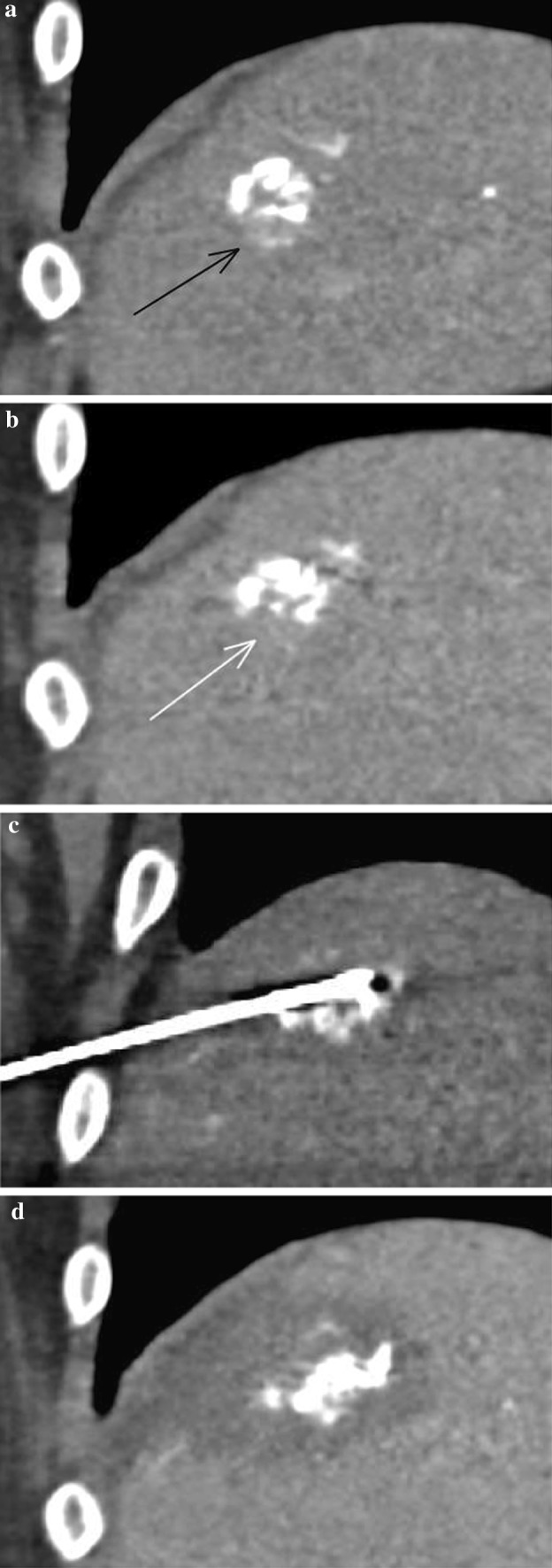
CT-guided ablation following TACE. Figure 3a, a coronal image from an arterial phase scan 18 months after the initial TACE, shows a small nodule of enhancement (black arrow) at the inferior aspect of the previously treated lesion, with washout on the venous phase (Fig. 3b, white arrow). The radiopaque beads allowed the lesion to be targeted for thermal ablation under CT guidance—Fig. 3c shows the microwave needle in the lesion. Figure 3d is from a venous phase scan one month later, showing satisfactory ablation margins around the radiopaque beads in the lesion

One patient in our cohort (Fig. 3) illustrates the value of radiopaque beads with respect to subsequent ablation. A 74-year-old male with a 3.7-cm segment 7 HCC, considered too big for primary ablation [3], underwent TACE with radiopaque beads. Follow-up imaging demonstrated complete response, with a reduction in lesion size to 12 mm, until a CT at 18 months showed a small (< 10 mm) area of recurrence at the lesion’s inferior aspect. With the disease now effectively down-staged following TACE, microwave ablation was performed with the radiopaque beads aiding needle placement under CT guidance. Follow-up CT one month later showed satisfactory ablation margins.

As well as ablation of recurrent or down-staged disease following TACE, there is increasing interest in the role of combining TACE and ablation as a primary treatment strategy, given that tumour necrosis induced by prior chemo-embolisation may result in better conduction of thermal energy and therefore more effective ablation [18, 19]. This approach was employed in four patients in our cohort, with radiopaque beads facilitating CT-guided ablation in three cases.

### Limitations

The retrospective design of the study, with no control group, means that the results do not reliably demonstrate the relative performance of radiopaque beads compared to more established products. Furthermore, it is problematic to compare our results with those of other studies given differences in patient selection, disease characteristics and embolisation strategy.

While radiopaque beads offer opportunities to explore correlations between bead distribution and clinical outcomes, which may help guide future developments in TACE techniques, such analysis is beyond the scope of this study in view of the small number of patients and variable follow-up period.

Given that data relating to complications were collected retrospectively from clinical records, we are likely to have under-estimated post-embolisation symptoms compared to others who conducted questionnaires for all patients [16].

## Conclusion

While the results of other studies evaluating TACE with DC Bead LUMI™ are awaited [20], we have demonstrated promising early clinical outcomes of this radiopaque bead. It ensures good intra- and post-operative visualisation and can facilitate subsequent CT-guided ablation in appropriate cases, either as a planned primary treatment strategy or for recurrent disease. Further evaluation will be required to optimise patient selection and establish conclusively the efficacy and safety of this new embolic agent.
